# Strategies for the Formation of Monolayers From Diazonium Salts: Unconventional Grafting Media, Unconventional Building Blocks

**DOI:** 10.3389/fchem.2020.00559

**Published:** 2020-07-14

**Authors:** Alice Mattiuzzi, Quentin Lenne, Janine Carvalho Padilha, Ludovic Troian-Gautier, Yann R. Leroux, Ivan Jabin, Corinne Lagrost

**Affiliations:** ^1^X4C, Gosselies, Belgium; ^2^Univ Rennes, CNRS, ISCR-UMR 6226, Rennes, France; ^3^Instituto Latino-Americano de Ciências da Vida e da Natureza, Universidade Federal da Integração Latino-Americana, Foz do Iguaçu, Brazil; ^4^Laboratoire de Chimie Organique, Université libre de Bruxelles (ULB), Brussels, Belgium

**Keywords:** diazonium, surface functionalization, monolayers, calixarene, ionic liquids

## Abstract

Pioneered by J. Pinson and coll. in 1990s, the reductive grafting of aryldiazonium salts has become a powerful method for surface functionalization. Highly robust interfaces result from this surface attachment, resistant to heat, chemical degradation and ultrasonication. Importantly, this approach can be applied to many materials, ranging from conducting, semi-conducting, oxides to insulating substrates. In addition, either massive, flat surfaces or nanomaterials can be functionalized. The method is easy to process and fast. The grafting process involves the formation of highly reactive aryl radicals able to attack the substrate. However, the generated radicals can also react with already-grafted aryl species, leading to the formation of loosely-packed polyaryl multilayer films, typically of 10–15 nm thick. It is thus highly challenging to control the vertical extension of the deposited layer and to form well-ordered monolayers from aryldiazonium salts. In this mini review, we briefly describe the different strategies that have been developed to prepare well-ordered monolayers. We especially focus on two strategies successfully used in our laboratories, namely the use of unconventional solvents, i.e., room temperature ionic liquids (RTILs), as grafting media and the use of calixarene macrocycles by taking benefit of their pre-organized structure. These strategies give large possibilities for the structuring of interfaces with the widest choice of materials and highlight the potential of aryldiazonium grafting as a competitive alternative to self-assembled monolayers (SAMs) of alkyl thiols.

## Introduction

Many objects of everyday life benefit from organic coatings for protective or cosmetic purposes. A strong bond between the material surface (carbon, metals, semi-conductors, glass, or polymers) and the organic layer could be necessary for some applications, e.g., biomedical items (stents, prostheses), small parts in precision watchmaking, implantable sensors and corrosion protection to name a few. Only few procedures are known to produce such strong bonds, and most of them are dependent on the type of surface. For instance, it is easier to derivatize hydrogenated silicon than carbon because the Si-H bond is not as strong (330–380 kJ.mol^−1^) as C(aromatic)-H (460 kJ.mol^−1^) (Fabre, 2016). Self-assembled monolayers (SAMs) of thiols represent a very popular strategy to form spontaneously chemisorbed layers on gold or copper surfaces but weak bonds are obtained with other industrial metals such as iron or nickel, limiting the practical use of such procedure (Love et al., 2005). The reduction of aryldiazonium salts allows a very strong attachment of aryl groups on surfaces (Pinson and Podvorica, 2005; Bélanger and Pinson, 2011), with adsorption energies of aryl groups close or above 200 kJ.mol^−1^ (Jiang et al., 2006a,b). Experimentally, the resulting interface is found resistant to heat, chemical degradation and ultrasonication. Moreover, the procedure can be applied to a wide range of materials including conducting (Au, Ni, carbon in all forms, Pt, Cu, Fe, Zn, stainless steel, etc.), semi-conducting (Si, SiGe, Ge, GaAS, etc.), oxides (ITO, TiO_2_, SnO_2_, SiO_2_, Fe_2_O_3_, etc.), and even insulating (glass, PMMA, PET, PP, etc.) substrates, being either flat surfaces or nanomaterials (Bélanger and Pinson, 2011; Mohamed et al., 2015). Diazonium salts can be easily obtained from aniline precursors and their half-life in solution can reach 5 days, provided that the pH is kept below 2–3 (Pinson and Podvorica, 2005). A wide array of substituents can be attached to the aromatic ring of the aniline precursors, offering a large choice for surface functionalization.

The grafting with aryldiazonium salts is commonly carried out in aqueous acid or aprotic organic solvents (ACN, DMF). Many different techniques can be employed to activate the reaction, including electrochemistry, reduction by chemical agents, photochemistry, ultrasonication, heating, microwave or spontaneous grafting with the surface acting as the reducing agent (Bélanger and Pinson, 2011). The grafting mechanism is obviously dependent of the experimental conditions. However, it is generally admitted that the grafting process starts by the concerted formation of an aryl radical and departure of dinitrogen upon reduction. The produced radical is able to bind to the surface. Because of their high reactivity, aryl radicals can either react with the surface of the material or with already grafted moieties. As a consequence, loosely-packed multilayers are generally formed with thickness typically in the 10–15 nm range. A second pathway contributing to the multilayers formation is also possible where the already grafted moiety reacts with an aryldiazonium cation, forming azo linkages (Pinson and Podvorica, 2005; Bélanger and Pinson, 2011).

Control of the vertical extension to form well-ordered structures, as those typically obtained with SAMs, is very challenging using diazonium salts. The development of strategies to accommodate the robustness of the diazonium grafting procedure with the preparation of well-organized monolayers has then aroused great interest in the community. Monolayers are of primary importance for some applications. In electrocatalysis or in electrochemical sensing a fast (electronic) communication is required between the sensitive layer and the analyte or compound to activate. In case of surface sensitive characterization (e.g., SPR or QCM techniques), the unambiguous detection of sensor signal is related to the interfacial molecular organization. Hence, this requires to keep the roughness as small as possible. Starting from a monolayer, it is possible to build well-organized functional interfaces for a dedicated application in a controlled manner using for instance chemical coupling with the organic layer (post-functionalization). Over the last 15 years, different research groups have developed approaches for controlling the grafting from aryldiazonium salts toward monolayers (Liu et al., 2007; Breton and Downard, 2017; Hapiot et al., 2018). A strict control of the experimental conditions (diazonium concentration, electrolysis time, applied potential) allows, in principle, the formation of monolayers, but generally leads to sparse (sub-)monolayers with poor reproducibility. Chemical engineering has provided the most robust strategies by taking benefit of the steric hindrance of bulky-protecting groups carried by the aryldiazonium (Nielsen et al., 2007; Combellas et al., 2008; Malmos et al., 2009; Leroux et al., 2010; Leroux and Hapiot, 2013; Lee et al., 2014, 2016). In some cases, the bulky-protecting groups could be removed, leaving a monolayer with reactive-*termini* groups available for further post-functionalization (Nielsen et al., 2007; Malmos et al., 2009; Leroux et al., 2010; Leroux and Hapiot, 2013; Lee et al., 2014). Another approach developed by Breton and co-workers uses a radical scavenger as a redox mediator to limit the growth of the layer (López et al., 2018).

Herein, we focus on two other strategies that rely on the use of room temperature ionic liquids (RTILs) as grafting media on the one hand and on the use of pre-organized calix[4]arene macrocycles as diazonium precursors on the other hand. We will then show that these two strategies have proved particularly suitable for functionalizing nanomaterials.

## RTILs as Grafting Media

**Figure 1:**
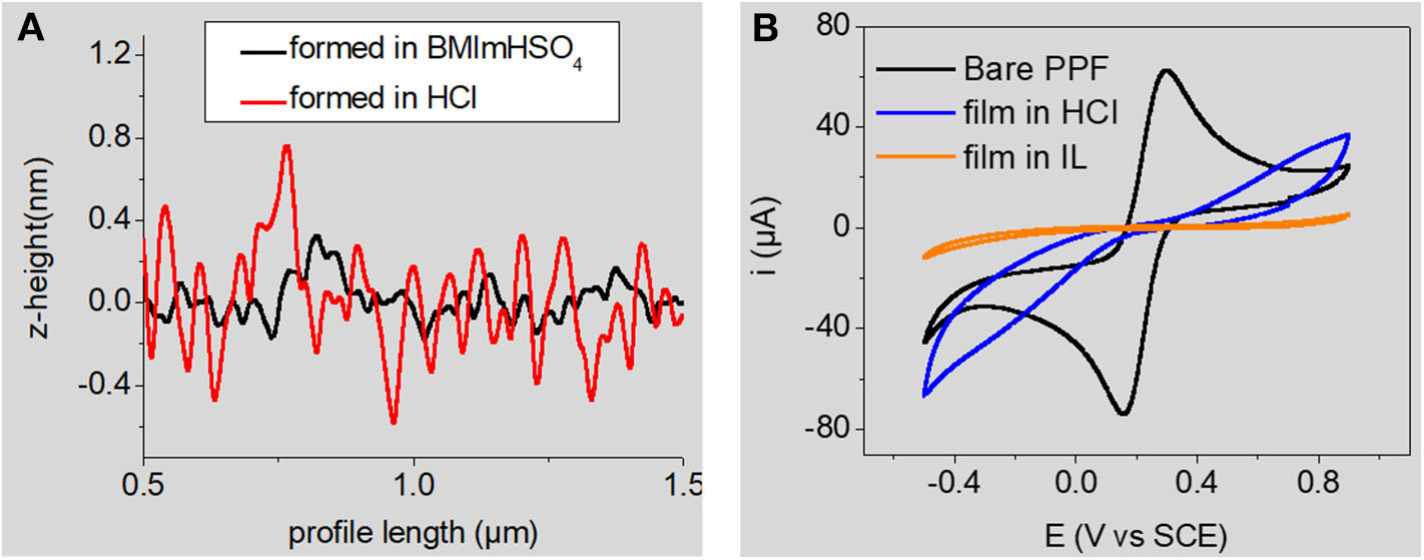
**(A)** AFM roughness profile of nitrobenzene layers electrografted in [BMIm][HSO_4_] (black line) and in aqueous 0.1 M HCl (red line) **(B)** cyclic voltammetry of 2 × 10^−3^ M K_4_Fe(CN)_6_ in aqueous 1M KCl at the same electrografted layers. Signal at the bare substrate is given for comparisons purposes. A full inhibition of the electrochemical signal of the redox probe is observed at the film formed in the RTIL Adapted from reference (Carvalho Padilha et al., 2016) Copyright Wiley-VCH Verlag GmbH & Co. KGaA. Reproduced with permission.

RTILs are salts that are liquid at room temperature. They generally consist of the association of a bulky asymmetric organic cation (imidazolium, pyrrolidinium, piperidinium, pyridinium, etc.) with a weakly coordinating anion (${\text{PF}}_{6}^{-}$, ${\text{BF}}_{4}^{-}$, bistriflimide, tosylate, etc.). RTILs have appealing physicochemical properties (thermal and chemical stabilities, redox-robustness, good ionic conductivity, very good solubilizing properties, negligible volatility, etc.) that can be easily tuned for a dedicated application. They have been widely investigated as electrolyte media for various electrochemical processes (Hapiot and Lagrost, 2008), notably electrodeposition of polymers. Very logically, they were also studied as grafting media for the electroreduction of aryldiazonium cations at glassy carbon electrodes (Actis et al., 2008; Ghilane et al., 2008; Fontaine et al., 2010; Shul et al., 2013; Carvalho Padilha et al., 2016). All of these reports point toward the formation of ultrathin layers, close to monolayers. Interestingly, Ghilane and Randriahamazaka have found a correlation through AFM measurements between the decrease of layer thickness and the increase in ionic liquids' viscosity (Fontaine et al., 2010). They studied the electrografting of 4-nitrobenzenediazonium cations in three hydrophobic ionic liquids and concluded that the use of ionic liquids led to the formation of a thinner and/or less dense layer compared to classical organic solvents (Fontaine et al., 2010). Using an acidic Brønsted ionic liquid, 1-butyl-3-methyl-imidazolium hydrogensulfate, [BMIm][HSO_4_], we have demonstrated that this ionic liquid promotes the formation of both denser and thinner layers compared to the classical acidic aqueous 0.5 M HCl medium (Carvalho Padilha et al., 2016). The acidic ionic liquid allows the *in situ* diazotization of 4-nitroaniline to produce the corresponding nitrobenzenediazonium cations in the presence of sodium nitrite. We observed an efficient self-patching and self-limiting growth of the grafted layer in this medium, whatever the charge consumed during the electrografting process (Carvalho Padilha et al., 2016). This result is interesting since the larger the charge, the thicker the layer is expected (Pinson and Podvorica, 2005). Our observation was in fair agreement with an earlier report by Bélanger and coll. who employed a protic ionic liquid consisting of a 1:2 mixture of 4-methoxypyridine with trifluoroacetic acid for the *in situ* production and electrografting of 4-nitrobenzene- and 4-chlorobenzenediazonium cations (Shul et al., 2013). The compactness of the layers prepared in [BMIm][HSO_4_] and in aqueous 0.1 M HCl was compared using AFM roughness profiling and by evaluating their blocking properties toward a redox probe (Figure 1), showing that layers formed in [BMIm][HSO_4_] are much more compact.

The high viscosity of [BMIm][HSO_4_] (900 mPa.s) may explain the very good compactness of the grafted layers by slowing down diffusion processes and hence limiting the diffusion of electrogenerated aryl radicals away from the electrode. However, viscosity is unable to explain the controlled formation of monolayers. The slower diffusion of aryl radical in the highly viscous RTILs should intuitively promote the formation of thicker layers because the radicals are much less prone to quickly diffuse away from the electrode surface. Hence, they should rapidly react in a confined volume at the vicinity of the electrode, logically forming thicker layers. As a consequence, other phenomena should be invoked, for instance the specific solvation and ordering of the interface between the electrode and RTILs. Because of the larger size of the ions, the absence of molecular solvent screening the charges and the ability of ionic liquids to endure a structural ordering through organized networks of anions and cations, the interface is likely to be strongly different from that obtained in aqueous or organic electrolytes (Kirchner et al., 2013; Fedorov and Kornyshev, 2014; Zhong et al., 2014). The presence of large ions, possibly ordered at the electrode interface, and as solvation shell surrounding the diazonium cations might impede the attack of aryl radicals to the already grafted moieties, inhibiting the growth of the layer.

## Calix[4]arene-Diazonium Salts

**Figure 2:**
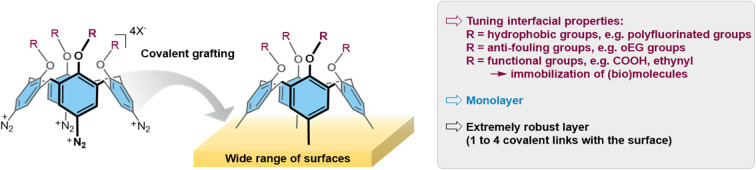
Strategy and key benefits for the grafting of calix[4]arene tetradiazonium salts.

Calix[4]arenes are macrocycles built from the linkage of four aromatic subunits through methylene bridges. With adequate groups at the small rim, they offer a rigid cone-constrained structure, being smart building blocks for the construction of highly robust monolayers with diazonium chemistry (Figure 2): (i) the methylene bridges prevent side reactions from the aryl radical and thus the formation of multilayers (ii) appending arms at the small rim allow the introduction of various functional molecules or objects with a fine spatial control imposed by the small rim geometry and (iii) several diazonium functions at the large rim provide multiple anchoring points, which is expected to enhance the stability of the monolayers as already highlighted in self-assembled monolayers (Li et al., 2019). We have thus functionalized the large rim of calix[4]arenes with diazonium functions, and densely-packed monolayers were obtained through the reduction of these calix[4]arene-based diazonium cations as revealed by AFM and ellipsometry measurements (Mattiuzzi et al., 2012; Troian-Gautier et al., 2020). A strong blocking effect toward redox probes, namely dopamine, is observed in agreement with the formation of compact layers with surface coverage of 79% (Mattiuzzi et al., 2012). Thus, a regular and smooth distribution of the macrocycles is obtained on the surface, making the structuring of the interface at the molecular scale possible. A closely-related work has been later described by another group where both large and small rims are equipped with anchoring functions (diazonium and ethynyl groups, respectively), leading to so-called Janus calix[4]arenes also useful for preparing monolayers (Buttress et al., 2016). The calixarene diazonium strategy has been developed using different substrates, either conducting [glassy carbon, PPF, gold (Mattiuzzi et al., 2012)] or semi-conducting [germanium (Blond et al., 2018)]. By using the spontaneous formation of radicals through decomposition of aryldiazonium salts in basic media, a layer of calix[4]arenes is even readily grafted onto polymers as demonstrated by AFM and XPS analyses (Troian-Gautier et al., 2016a). All these works exemplify the general character of the strategy which can be applied to the widest range of materials.

A very interesting point is the possibility to vary the functions at the small rim for tuning interfacial properties. For instance, polyfluorinated groups decorating the small rim bring robust interfacial hydrophobic properties to gold, polypropylene and glass substrates (Mattiuzzi et al., 2020). Water contact angles >110° were measured for the coated substrates while the bare pristine glass, gold and polypropylene surfaces were characterized by contact angles of 24.6°, 64.7°, and 102.9°, respectively. Importantly, these hydrophobic nanometric interfaces resist harsh washing and aging. Monolayers of calix[4]arenes bearing oligo-(ethylene glycol) (oEG) chains grafted on germanium and gold surfaces exhibit antifouling properties toward a protein, BSA (Blond et al., 2018). Post-functionalization through chemical coupling “on-surface” is another very effective way to introduce new interfacial properties thanks to molecules with dedicated *termini* or able to execute functions under stimuli (pH variation, electron transfer, etc.). Reactive pendant groups at the calix[4]arenes small rim such as carboxylic acid or ethynyl were successfully post-functionalized (Mattiuzzi et al., 2012; Santos et al., 2014; De Leener et al., 2016; Troian-Gautier et al., 2016a). For example, amide coupling reaction with ferrocenemethylamine allowed the attachment of ferrocene redox units to monolayers of calix[4]arenes displaying one or four carboxylic acid reactive *termini* (Mattiuzzi et al., 2012). The surface concentration of ferrocene coupled to the monolayers has been determined through electrochemical measurements. With one reactive pendant group, the surface concentration of ferrocene actually corresponds to the surface concentration of calix[4]arene itself, and the value found (Γ = 6.9 ± 0.6 × 10^−11^ mol.cm^−2^) further confirms the formation of a compact monolayer since the calculated theoretical value for a close-compact monolayer is 9.8 × 10^−11^ mol.cm^−2^ (Mattiuzzi et al., 2012). Interestingly, the surface concentration with monolayers carrying four reactive pendant groups was found twice as high, indicating that two ferrocene units could be coupled to each calixarene, in fair agreement with the steric crowding of ferrocenemethylamine with respect to the geometry of the small rim (Mattiuzzi et al., 2012). This result demonstrates the preparation of functionalized surfaces with a fine spatial control between coupled units imposed by the geometry of small rim.

Lastly, the one-pot grafting from mixture of calix[4]arene-tetradiazonium salts with different functionalization patterns at the small rim leads to the controlled formation of mixed monolayers (Santos et al., 2014). Preparation of binary mixed layers is particularly challenging with diazonium grafting because of the high and unselective reactivity of the produced aryl radicals (Jiang et al., 2017). Generally, the surface concentration of the easiest to reduce component is always higher than that of the other component, whatever its mole fraction in the grafting medium. Thanks to the common macrocyclic scaffold that sterically isolates the diazonium groups from the other functional groups, the different calixarenes-tetradiazonium salts exhibit close reduction potentials. In addition, the covalent nature of the grafting limits the segregation effect, often observed with SAMs of thiols. Wettability studies and scanning electrochemical analyses show that the ratio of each calixarene component on the surface agrees well with that of the diazonium salts in the deposition solution (Santos et al., 2014). Thus, the variation of contact angles of the modified surfaces with the mole fraction of each calixarene-tetradiazonium cations in solution follows an Israelachvili's equation (Israelachvili and Gee, 1989), indicating that the mixing is effective at very small length scales. Israelachvili's equation is a quadratic relationship between the equilibrium contact angle and molar fraction of a layer's component that derives from Cassie's model (Cassie and Baxter, 1944). Israelachvili's model assumes a chemical heterogeneity of molecular dimensions (Israelachvili and Gee, 1989) while Cassie's model applies to surfaces with separate, discrete chemical patches (Cassie and Baxter, 1944).

## Nanomaterials

The two strategies described in the previous sections were successfully transferred to nanomaterials and were particularly relevant for their functionalization.

The use of acidic Brønsted ionic liquid as grafting medium was extended to the functionalization of carbon nanotubes. As early as 2005, Tour and co-workers have employed imidazolium-based ionic liquids for exfoliating and functionalizing single-walled carbon nanotubes as individuals, by simply grinding carbon nanotubes with aryldiazonium salts in the presence of ionic liquids and K_2_CO_3_ (Price et al., 2005). More importantly, imidazolium-based ionic liquids are capable of forming bucky gels with 0.1–1 wt% of carbon nanotubes (Fukushima and Aida, 2007). These bucky gels are highly stable as opposed to classical organogels or hydrogels, and the dispersion ability of the ionic liquid is attributed to cation-π interaction between the imidazolium and the surface of carbon nanotubes. We took benefit of this property to functionalize the sidewalls of single and multi-walled carbon nanotubes (Carvalho Padilha et al., 2016). The approach shows high versatility, allowing both electrochemical and chemical grafting of carbon nanotubes with aryldiazonium cations in [BMIm][HSO_4_].

The covalent grafting of calix[4]arene derivatives was also developed on gold nanoparticles, notably with calix[4]arenes bearing carboxylic acid groups at the small rim (Troian-Gautier et al., 2016b). Two pathways were implemented, either a one-step route consisting in the simultaneous reduction of diazonium cations and gold salts or a two-step route with commercially available nanoparticles that underwent a ligand exchange reaction. Both routes are efficient, and notably the first one leads to monodisperse spherical nanoparticles of ~6 nm. The key point is the exceptional colloidal stability of the resulting nanoparticles in aqueous solutions. In strong contrast with gold nanoparticles capped with citrate or thiols that are usually sensitive to pH or ionic strength changes, the nanoparticles functionalized with calix[4]arenes could be reversibly suspended and precipitated through pH variations from 0.75 to 13, but also dried and then resuspended (Troian-Gautier et al., 2016b). Similar results have been reported for gold nanoparticles coated with long polymer chain (Manson et al., 2011), but the observed resuspendability with calixarene-coated nanoparticles is really remarkable for a capping layer of only a few nanometer thickness. Moreover, by taking benefit of reactive carboxylic acid pendant groups, the gold nanoparticles could be easily post-functionalized, e.g., with dodecylamine. The as-modified nanoparticles were then rendered soluble in organic solvents such as diethylether (Troian-Gautier et al., 2016b). Lastly, mixed layers of two calix[4]arenes can be grafted onto AuNPs in controlled ratio (Valkenier et al., 2017).

## Conclusions

Many efforts have been devoted to the controlled formation of monolayers with diazonium grafting. The use of ionic liquids as a solvent is very interesting but requires to clarify their specific effects in limiting the growth of the layers. The calix[4]arene strategy is a very reliable and versatile approach for forming dense and compact monolayers with reactive appending arms and/or controlled composition. Due to the high robustness of the interface, the easy synthesis and the wide choice of surface materials, this approach may even surpass the classical SAMs of thiol derivatives through a mild molecular route, and can bring significant advances for tailoring surfaces for (bio)sensing and electrocatalysis.

## Author Contributions

JC and CL contributed to the work in ionic liquids. AM, QL, LT-G, YL, IJ, and CL contributed to the work with calixarenes. The manuscript was written through contributions of all authors.

## Conflict of Interest

AM is currently employed by the company X4C. LT-G was employed by the company X4C. IJ, AM, and CL are shareholders of X4C. IJ is a consultant for X4C. The remaining authors declare that the research was conducted in the absence of any commercial or financial relationships that could be construed as a potential conflict of interest.
